# Suppurative Appendicitis—Operation—Recovery

**Published:** 1893-06

**Authors:** Edward M. Albers

**Affiliations:** Victoria, Texas


For Daniel's Texas Medical Journal.

### SUPPURATIVE APPENDICITIS — OPERATION—RECOVERY.

BY EDWARD M. ALBERS, M. D., VICTORIA, TEXAS.

GEO. H. White, male, 23 years of age. Well developed, weighing 185 pounds. Taken violently ill on the 12th of February, 1893, after eating some almonds, which he had not thoroughly masticated; abdominal pain marked. Called in a physician who administered morphia hypodermically, and mercurial purge; continued the administration of morphia until the evening of the 15th inst., when he discharged his then medical attendant and called me in. Found him narcotized, temperture 105°, pulse thready, but not over 100. Tongue thickly coated, gums swollen and tender, breath offensive, no action on bowels since the 12th, a countenance indicating anxiety and marked depression; complaining of great pain and tenderness all over the abdomen, especially marked in the right illiac region. By careful palpation and percussion outlined a tumefaction, occupying the right iliac region, and extending up to or near the lower border of the ribs. Pressure over McBurney's point elicited pain, marked upon firm pressure. Diagnosed the case appendicitis, and determined not to mask the symptoms with morphia; accordingly withdrew morphia, and put him on quiniæ sulp. gr. v, every three hours, ammon. carb. 10 gr. and ℥iv whisky every three hours, rectal injections of hot water (hot as could be borne), to be repeated every four hours,—which promptly emptied the rectum and afforded marked relief. On following morning, 16th inst., found him much improved, temp. 99½°, pulse fuller, tenderness considerably diminished. On the following day he refused to use the hot water injections, owing to some fancied cause;—continued to improve until the 19th. On the evening of the 18th prescribed a saline purgative, during the night it operated two or three times; in the morning found him with a temperature of 102° and thready pulse. He stated that during the nigh the awoke feeling greatly depressed. Concluded perforation had taken place, and an operation demanded; called another physician in consultation, who advised delay, which was consented to. Temperature for the next three days ranged from 102° in the morning to 104° in the evening. wlth pulse increasing in frequency. On the morning of the 22nd, sudden and alarming symptoms of pulmonary complication developed—resp. 36 to 40, dullness on percussion on the right chest, extending above the nipple, complete absence of respiratory sounds, and considerable cough with blood streaked expectoration. Decided to operate at once. Ably assisted by Drs. Duncan and Thornton, I operated under chloroform and full antiseptic measures; made a careful dissection from McBurney's point in an oblique direction three or four inches to the right. Upon entering the cavity thick and fetid pus poured out, some two or three pints, some fecal matter mixed with the pus. After exploring the cavity, inserted a large rubber drainage tube, and packed iodoform gauze over tube and incision; did not wash out the cavity. Marked improvement followed the operation; temperature in a few hours declined to normal with a corresponding improvement in the pulse, and a subsidence at once of the pulmonary symptoms. The discharge of pus continued offensive for seven or eight days; moved bowels by glycerine enema three days after operation, inability to evacuate bladder for one week after operation; necessitating use of the catheter; after that period function returned. Removed the tube twenty-six days after operation, discharged the patient cured on the 1st of April. He has regained his weight and resumed his occupation of livery-stable man.

There are several points I wish to call especial attention to in this case. The apparent improvement after using the hot injections.

Was the saline purgative responsible for causing perforation by increasing peristalsis?

Did not wash out or pack cavity, but kept external parts clean, and used iodoform gauze over tube with an outside layer of bichloride of mercury gauze. And lastly the pulmonary complication. I find no mention of this by authors on appendicitis. My own conclusion was, the liver displaced upwards by the tumefaction thereby incroaching upon the capacity of the lung.

In conclusion I should state nothing further could be desired in this case, as the convalescence was prompt and progressive from the hour of operation.

